# Comparison Between High- and Low-Intensity Static Stretching Training Program on Active and Passive Properties of Plantar Flexors

**DOI:** 10.3389/fphys.2021.796497

**Published:** 2021-12-17

**Authors:** Masatoshi Nakamura, Riku Yoshida, Shigeru Sato, Kaoru Yahata, Yuta Murakami, Kazuki Kasahara, Taizan Fukaya, Kosuke Takeuchi, João Pedro Nunes, Andreas Konrad

**Affiliations:** ^1^Institute for Human Movement and Medical Sciences, Niigata University of Health and Welfare, Niigata, Japan; ^2^Department of Physical Therapy, Niigata University of Health and Welfare, Niigata, Japan; ^3^Department of Rehabilitation, Matsumura General Hospital, Iwaki, Japan; ^4^Department of Rehabilitation, Kyoto Kujo Hospital, Kyoto, Japan; ^5^Department of Physical Therapy, Faculty of Rehabilitation, Kobe International University, Kobe, Japan; ^6^Metabolism, Nutrition, and Exercise Laboratory, Physical Education and Sport Center, Londrina State University, Londrina, Brazil; ^7^Institute of Human Movement Science, Sport and Health, University of Graz, Graz, Austria

**Keywords:** stretch training, resistance training, extended-field-of-view, ankle plantar flexors, muscle thickness, pennation angle, fascicle length

## Abstract

The purpose of this study was to compare two static stretching (SS) training programs at high-intensity (HI-SS) and low-intensity (LI-SS) on passive and active properties of the plantar flexor muscles. Forty healthy young men were randomly allocated into three groups: HI-SS intervention group (*n* = 14), LI-SS intervention group (*n* = 13), and non-intervention control group (*n* = 13). An 11-point numerical scale (0–10; none to very painful stretching) was used to determine SS intensity. HI-SS and LI-SS stretched at 6–7 and 0–1 intensities, respectively, both in 3 sets of 60 s, 3×/week, for 4 weeks. Dorsiflexion range of motion (ROM), gastrocnemius muscle stiffness, muscle strength, drop jump height, and muscle architecture were assessed before and after SS training program. The HI-SS group improved more than LI-SS in ROM (40 vs. 15%) and decreased muscle stiffness (−57 vs. −24%), while no significant change was observed for muscle strength, drop jump height, and muscle architecture in both groups. The control group presented no significant change in any variable. Performing HI-SS is more effective than LI-SS for increasing ROM and decreasing muscle stiffness of plantar flexor muscles following a 4-week training period in young men. However, SS may not increase muscle strength or hypertrophy, regardless of the stretching discomfort intensity.

## Introduction

Static stretching (SS) interventions are generally performed in sports and rehabilitation settings to improve a variety of fitness- and health-related capacities. In regard to muscle morphofunction, literature indicates that performing SS may increase joint range of motion (ROM) and stretch tolerance after minutes of exercise practice and weeks of training intervention, whereas the effects on muscle stiffness, performance, and architecture remain controversial (Nakamura et al., 2012; Konrad and Tilp, 2014; Medeiros and Lima, 2017; Nunes et al., 2020b). Among the factors that have explained the differences between results in the literature, the main one refers to the variety of characteristics of the SS programs; type, volume, and intensity of the stretching exercises.

Recently, the exercise intensity has been proposed as a major factor that affects the effectiveness of SS programs (Apostolopoulos et al., 2015; Nunes et al., 2020b). Previous studies focusing on the acute effects of stretching intensity indicate larger increases in ROM and decreases in passive stiffness of the hamstring muscles for high-intensity SS than normal intensity SS (Fukaya et al., 2020b; Takeuchi and Nakamura, 2020a; Takeuchi et al., 2021a,b,c). In addition, an acute study on gastrocnemii also showed that a short-duration high-intensity SS could decrease muscle stiffness to a greater extent than long-duration low-intensity SS (Fukaya et al., 2020a). Thus, high-intensity SS intervention seems to be effective in immediately increasing ROM and decreasing passive stiffness.

However, in contrast to these immediate acute effects, no benefits have been shown for higher intensities following bouts of SS on chronic adaptations (Magnusson et al., 1996; Folpp et al., 2006; Beltrão et al., 2020; Fukaya et al., 2020b). Specifically, Fukaya et al. (2020b) investigated two different stretching intensities for 4 weeks (100 vs. 120% of the hamstring muscles maximum tolerable ROM; 1 set of 60 s; 3×/week) and showed similar results on ROM between conditions and no change in passive stiffness in both conditions. In a longer duration study, Beltrão et al. (2020) compared the effects of SS performed at low (1–2 out of 10) and high intensities (9–10 out of 10; pain/discomfort intensity scale) on the hamstring muscles (3 sets of 60 s; 3×/week; 12 weeks). Results indicated adaptations of similar magnitudes on ROM and stretching tolerance for both groups, however, none showed changes in passive stiffness and muscle architecture. Therefore, there could be a discrepancy between the acute effect and chronic effect of high-intensity SS intervention on ROM and passive stiffness. This discrepancy might be related to differences in the outcome measurements. These previous studies investigated the chronic effect of a SS training program on passive torque or passive stiffness without considering the muscle elongation *via* ultrasound technique. However, these variables could reflect the stiffness of many tissues other than the muscle (e.g., ligaments and joint capsule) (Nojiri et al., 2019). In fact, Ichihashi et al. (2016) investigated effect of SS training program with normal intensity on muscle stiffness of the hamstring muscles and reported that muscle stiffness of all muscles comprising the hamstring muscles decreased. However, to the best of our knowledge, there is no study to compare the effects of SS training programs with different stretching intensities on muscle stiffness. Thus, it remains to be explored whether the SS intensity influences the muscle stiffness and whether a higher intensity above the discomfort, i.e., causing the pain, would present greater results after a SS training program.

Moreover, the effects of SS training programs on muscle performance and architecture remains controversial. Interestingly, previous studies that resulted in changes in these variables could adopt the high-intensity SS training program (Simpson et al., 2017; Mizuno, 2019; Nunes et al., 2020b). In fact, a high-intensity SS intervention could cause muscle strength and architecture changes due to the more intense stimuli on the muscle. To date there are no studies comparing the effects of SS training programs with different stretching intensities. Consequently, it is not clear whether differences in stretching intensity, especially high-intensity SS interventions, are important for muscle strength and architecture changes.

Therefore, the purpose of this study was to compare the effects of a SS intervention program at two different stretching intensities, i.e., high-intensity (HI-SS) vs. low-intensity (LI-SS), on passive and active properties of the plantar flexor muscles. Based on the acute effects recently analyzed on this muscle group (Fukaya et al., 2020a), we hypothesized that HI-SS would present larger increases in ROM and decreases in muscle stiffness than LI-SS. Also, we hypothesized that only HI-SS would change muscle strength and architecture (Nunes et al., 2020b).

## Materials and Methods

### Experimental Design

A randomized, repeated measures experimental design was used to investigate the chronic effects of high-intensity and normal-intensity SS intervention on plantar flexors muscle architecture (muscle thickness, pennation angle, and fascicle length), passive properties [ankle dorsiflexion ROM (DF ROM), passive torque, angle at first stretch sensation, and muscle stiffness], and muscle strength (maximal voluntary isometric and concentric contractions, and drop jump height) in the dominant leg (preferred to kick a ball). In both stretching intervention groups, all variables were measured before (PRE) and after (POST) a 4-week SS training program (Nakamura et al., 2012, 2017). To prevent any effect from the last session, all POST measurements were repeated at least 24 h after the final SS program session. The participants in both stretching intervention groups performed SS once a day, three times a week for four weeks, with at least a 24–48 h rest period between interventions, using a stretching board (Asahi stretching board, Asahi Corp., Gifu, Japan). A control (CON) group underwent PRE and POST measurements without any intervention. All participants were instructed to refrain from stretching, therapeutic massage, and/or resistance training outside of the study during their participation. The Ethics Committee of the Niigata University of Health and Welfare, Niigata, Japan (Procedure #17677), approved the study and we complied with the requirements of the Declaration of Helsinki.

### Participants

Forty healthy male volunteers participated in this study. The participants were randomly assigned to either a HI-SS group (*N* = 14, age, 21.4 ± 1.0 years; height, 172.2 ± 5.1 cm; weight 61.7 ± 6.1 kg), a LI-SS group (*N* = 13, age, 21.4 ± 1.1 years; height, 169.5 ± 5.6 cm; weight 64.4 ± 8.3 kg), or a CON group (*N* = 13, age, 21.9 ± 1.3 years; height, 170.5 ± 4.4 cm; weight 63.3 ± 4.8 kg). There were no significant differences in age, height, or body mass among the groups at baseline. As inclusion criteria, participants should present no history of neuromuscular disease and/or musculoskeletal injury involving the lower extremities, and not currently perform regular stretching and/or resistance training for their lower limbs. They were university students who were not team members of amateur or professional sports. All participants were fully informed of the procedures and purpose of the study, after which they provided written informed consent. We calculated the sample size required for a split-plot analysis of variance (ANOVA) (effect size = 0.69, alpha error = 0.05, power = 0.80) using G*power 3.1 software (Heinrich Heine University, Düsseldorf, Germany) as per a previous study (Fukaya et al., 2020a), and the requisite number of participants for this study was more than 11 in each group.

### Dorsiflexion Range of Motion and Passive Torque Assessment

The participants sat on a dynamometer chair with a 0° knee angle (i.e., the anatomical position) and adjustable belts were fixed over the trunk and pelvis (Biodex System 3.0, Biodex Medical Systems Inc., Shirley, NY, United States). The participants were reclined (70° hip angle; 0° full extension) to prevent tension at the back of the knee. The footplate of the dynamometer was passively and isokinetically dorsiflexed at a speed of 5°/s from the neutral anatomical position to the dorsiflexion angle just before participants started to feel discomfort or pain (Akagi and Takahashi, 2013; Nakamura et al., 2020; Sato et al., 2020). Before the passive dorsiflexion assessment, two cycles of passive dorsiflexion were performed to familiarize the participants and to prevent a conditioning effect of passive stretching on the muscle-tendon stiffness (Konrad and Tilp, 2014; Hirata et al., 2017; Nakamura et al., 2021b). In addition, investigators visually confirmed that there was no heel displacement during passive stretching. After familiarization trials, the participants stopped the dynamometer by activating a hand-held safety remote button when they started to feel discomfort or pain, and the angle just before this point was defined as the DF ROM. The measurement was performed twice, and the average value was used for analysis. In addition, passive torque at the DF ROM was defined as the stretch tolerance (Weppler and Magnusson, 2010; Mizuno et al., 2013). Passive torque and ankle angle were converted from analog to digital values at a sampling rate of 1000 Hz (PowerLab 16SP; PowerLab System, AD Instruments Pty Ltd., Australia).

The first stretching sensation (FSS) angle was defined as the first self-perceived stretch sensation during the DF ROM measurements. The participants were instructed to stop the passive movement at the angle of the FSS using the safety trigger (Krause et al., 2019). The FSS angle was measured twice, using the average value for analysis.

Throughout the passive *DF* test, participants were requested to relax completely and not to offer any voluntary contractions. We confirmed that there were no voluntary contractions of the medial gastrocnemius (MG) by monitoring muscle activity by surface electromyography (FA-DL-720-14 Assist, Tokyo, Japan). Surface electrodes (Blue Sensor N, Ambu A/S, Ballerup, Denmark) were placed on the muscle belly of the MG. We confirmed that all data was collected during a relaxed state, i.e., did not show muscle activity exceeding 5% of maximal voluntary isometric contraction (MVC-ISO) (Nakamura et al., 2011).

### Muscle Stiffness Assessment of the Medial Gastrocnemius

A B-mode ultrasound imaging device (LOGIQ e V2; GE Healthcare Japan, Tokyo, Japan) and an 8-MHz linear array probe (imaging frequency: 56 Hz) were used to assess elongation of the MG during the passive *DF* test. We obtained longitudinal ultrasound images of the MG, which were synchronized to the passive torque and joint angle outputs. The ultrasound probe was placed on the distal MG near the muscle-tendon junction (MTJ). The ultrasound probe was secured with a standard orthopedic stocking to prevent movement of the probe during the passive dorsiflexion test. In POST measurement, ultrasound images were obtained and compared to images acquired at PRE to assure the same position of measurement. An acoustically reflective marker was placed on the skin under the ultrasound probe proximal to the MTJ of the MG to verify that the probe remained stable during the measurement (Morse et al., 2008; Nakamura et al., 2011). Ultrasound MTJ images were quantified using open-source digital measurement software (Image J, National Institutes of Health, Bethesda, MD, United States). MTJ displacement was defined as the distance between the MTJ and the reflective marker.

The muscle force of the MG was estimated by multiplying the measured passive torque by the relative contribution of the physiological cross-sectional area (18%) of the MG within the plantar flexor muscles (Kubo et al., 2002; Konrad et al., 2017) and then dividing it by the moment arm of the triceps surae muscle which was determined to be the length of the triceps surae muscles at a neutral position (90 degrees) of the ankle (50 mm) (Kubo and Ikebukuro, 2019). Passive muscle stiffness (N/mm) was calculated as the change in the passive torque from the neutral ankle position (0°) to DF ROM (smallest angle between PRE and POST) and divided by the MTJ displacement (Nakamura et al., 2021b). The previous studies (Hirata et al., 2015; Nakamura et al., 2021a) showed that the slack angle of MG was the plantar flexion position. Thus, the range used in this study could be beyond the point disappeared the slack of MG and the linear portion of MG.

### Maximal Voluntary Isometric Contraction Measurements

Participants were seated in the isokinetic dynamometer chair at a 0° knee angle (the anatomical position = knee extended) with adjustable belts fixed over their trunk and pelvis. Participants were reclined (70° hip angle; 0° = full extension) to prevent tension in the posterior knee. The trunk and pelvis were firmly fixed with straps, and trunk movement was restricted by holding the handle with both hands. The MVC-ISO of the plantar flexor was measured with the ankle joint at 30° plantar flexion and at the neutral (neutral anatomical position = 0°), at 15° dorsiflexion positions. To obtain accurate measurements, the ankle joint of the tested leg was securely attached to the footplate of the dynamometer using a velcro strap. A soft cloth was inserted between the velcro strap and instep to prevent movement of the ankle joint. After several warm-up submaximal plantar flexion contractions, two MVC-ISOs were performed for 3 s at each ankle position with 60-s intervals. The subjects were instructed to perform plantar flexion as fast and as hard as possible at the established position, for about 3 s. The average value of two MVC-ISO was used for analyses in each angle. Strong verbal encouragement was provided to promote participants’ maximal effort during contractions.

### Maximal Voluntary Concentric Contraction Measurements

The concentric muscle strength of plantar flexors was measured in the same position as that in the MVC-ISO measurements. ROM was from 10° of dorsiflexion to 20° of plantar flexion, with an angular velocity of 30 and 120°/s. Additionally, concentric contraction protocols were applied five times in each sequence. Throughout the measurement, participants were verbally encouraged during muscle contraction to promote maximal efforts. Maximum torque was measured during both concentric contraction velocities.

### Single-Leg Drop Jump Height

Single-leg drop jumps were performed from a 20-cm box onto a set of mat switches (Jump mat system; 4Assist, Tokyo, Japan). After three familiarization repetitions, three sets of single-leg drop jumps were performed and measured. Participants were instructed to step off the box for single-leg drop jump measurements and, upon landing with the same leg, immediately perform a maximal vertical jump using only the dominant side of the ankle plantar flexors without trying to minimize the use of the knee and hip muscles. We also ensured that the knee and hip joints moved as little as possible during the jump measurement. Both hands were crossed in front of the chest. The maximal vertical jump height over three jump measurements was then calculated using the flight time method.

### Muscle Thickness, Pennation Angle, and Fascicle Length

Participants were instructed to lie relaxed on a treatment table in a prone position with the hip and knee angle at 0° with the ankle angle at a slight plantar flexion. B-mode ultrasonography (LOGIQ e V2; GE Healthcare Japan, Tokyo, Japan) with an 8 MHz linear array probe was used to evaluate the muscle thickness and the pennation angle of the medial and lateral gastrocnemius muscles (MG and LG, respectively). Longitudinal ultrasound images were obtained for the MG and LG at 30% of the lower-leg length, measured from the popliteal crease to the lateral malleolus near the point of the maximal cross-sectional area of the lower leg (Akagi and Takahashi, 2013; Nakamura et al., 2014; Nunes et al., 2020a). Additionally, a longitudinal ultrasound image of the soleus muscle was obtained at 50% of the lower-leg length (Kubo et al., 2014, 2017). Muscle thickness of the MG, LG, and soleus and pennation angle of the MG and LG were determined by the measurement function of the ultrasonography system. Muscle thickness was determined as the mean of the distances between the deep and superficial aponeuroses measured at both ends of each image (Ema et al., 2013; Yahata et al., 2021). Additionally, the pennation angle was determined as the mean of the three fascicles at the angle between the fascicle and deep aponeurosis.

In this study, we used a technique called extended-field-of-view, and the fascicles of the MG and LG were defined as the distance between the origin of the fascicle on the superficial aponeurosis to its insertion on the deep aponeurosis. In this study, three different fascicles in the MG and LG were measured, and the average values were used for further analysis.

### High- and Low-Intensity Static Stretching Training Programs

Participants were instructed to perform a stretching program on the intervention side for 4 weeks using a stretching board similar to previous studies (Akagi and Takahashi, 2013, 2014; Nakamura et al., 2021b; Yahata et al., 2021). The stretching intensity was based on the 11-point Verbal Numerical Scale, being 0 as “not pain at all,” and 10 as “very, very painful.” In the HI-SS group, the stretching intensity was defined as between 6 and 7, and in the LI-SS group, the stretching intensity was defined as between 0 and 1, which was defined as the greatest tolerated dorsiflexion angle without pain or with little pain. Participants were instructed to move their body mass forward when deemed necessary to achieve the intensity required. All stretching intervention sessions were performed in a laboratory under the direct supervision of the research team where the study was conducted. The stretching intervention included 3 sets of 60 s, with 30 s intervals (Santos et al., 2020). The stretching intervention program was performed 3 days/week for 4 weeks at 1–2 day intervals (12 sessions). All SS sessions were performed in the laboratory under the direct supervision of the research team. The stretching intensity was modified between and within the stretching sessions *via* adjustment of the angle of the stretching board.

### Test-Retest Reliability of the Measurements

Test-retest reliability was assessed by the coefficient variation (CV) and the intraclass correlation coefficient (ICC) using 7 healthy men (22.7 ± 1.3 years, 170.4 ± 5.3 cm, 61.8 ± 4.4 kg) with 1 week between the two measures without any intervention. The CV and ICC of the measurements are shown in Table 1. The ICC ranged from 0.852 to 0.976, and CV ranged from 1.3 to 8.5%.

**TABLE 1:** The test and retest reliability of passive dorsiflexion range of motion (DF ROM), passive torque at DF ROM, first stretch sensation, muscle stiffness, maximal voluntary isometric contraction torque of plantar flexors (MVC-ISO) at three different positions, maximal voluntary concentric contraction torque (MVC-CON) at 30 and 120°/s and drop jump height.

	ICC (1, 1)	CV (%)
DF ROM	0.905	7.7 ± 9.6
passive torque at DF ROM	0.926	4.4 ± 5.5
first stretch sensation	0.948	8.2 ± 7.3
muscle stiffness	0.960	8.5 ± 6.6
MVC-ISO at 30° plantar flexion	0.951	5.0 ± 3.6
MVC-ISO at neutral position	0.942	2.4 ± 2.5
MVC-ISO at 15° dorsiflexion	0.979	1.3 ± 0.6
MVC-CON at 30°/s	0.914	2.3 ± 1.7
MVC-CON at 120°/s	0.852	5.8 ± 3.8
drop jump height	0.863	4.6 ± 1.6

*CV, coefficient variation; ICC, intraclass correlation coefficient.*

### Statistical Analyses

SPSS version 25.0 (IBM Corp., Armonk, NY, United States) was used to conduct statistical analyses. The normal distribution of the data was confirmed using the Shapiro–Wilk test. Differences in all variables at PRE assessment among each group were investigated using one-way ANOVA. For all variables, a split-plot ANOVA using two factors [time (PRE vs. POST assessment) and group (HI-SS group vs. LI-SS group vs. Control group)] was used to determine interaction and main effects. If the interaction effect was significant, a *post hoc* analysis was conducted using a paired *t*-test on each group to determine differences between PRE and POST values. The relative changes (Δ%) in the variables from PRE to POST were also calculated, and comparisons between the HI- and LI-SS groups were performed by a Mann-Whitney *U* test. Effect size (ES) was calculated as the mean difference between PRE and POST divided by pooled pre-training SD (Cohen, 1988). An ES of 0.00–0.19 was considered trivial, 0.20–0.49 was small, 0.50–0.79 was moderate, and ≥0.80 was large. The Spearman’s rank correlation coefficients (rs) were computed to quantify the relationship between PRE and POST measurements of DF ROM and passive torque at DF ROM or muscle stiffness in each HI- and LI-SS group. Statistical significance was defined as *p* < 0.05. Descriptive data were reported as mean ± SD.

## Results

### Comparison of the PRE Assessment Values Among the Three Groups

The one-way ANOVA showed that no differences were found for any variables among the groups.

### Dorsiflexion Range of Motion, Passive Torque at Dorsiflexion Range of Motion, and First Stretching Sensation

Changes in DF ROM, passive torque at DF ROM, and FSS after the 4-week regular SS intervention program are shown in Table 2. The split-plot ANOVA showed interaction effects and main effects of time for the DF ROM, passive torque at the DF ROM, and muscle stiffness, whereas there was no significant interaction effect and the main effect of time for FSS. The *post hoc* test showed that the DF ROM was increased in both HI-SS and LI-SS groups (*p* < 0.01, *d* = 0.92; *p* = 0.03, *d* = 0.33), and the relative change (Δ%) in the DF ROM in the HI-SS group (Δ% = 46.5 ± 35.8) was significantly higher than the LI-SS group (Δ% = 15.8 ± 15.5, *p* = 0.028, *d* = 1.20). Passive torque at the DF ROM was significantly increased only in the HI-SS group (*p* = 0.019, *d* = 0.65). Muscle stiffness was significantly decreased in both HI-SS and LI -SS groups (*p* < 0.01, *d* = 1.19; *p* = 0.04, *d* = 0.30), and the relative change in muscle stiffness in the HI-SS group (Δ% = −55.0 ± 13.9) was significantly higher than the LI-SS group (Δ% = −22.2 ± 14.0, *p* < 0.01, *d* = 2.35). No significant changes in any variables were observed for the control group.

**TABLE 2:** Changes (mean ± SD) in dorsiflexion range of motion (DF ROM), passive torque at DF ROM, first stretch sensation, and muscle stiffness before (PRE) and after (POST) 4-week static stretching program at high-intensity (HI) or low-intensity (LI), and control group without stretching intervention.

	HI stretching group		LI stretching group		Control group		ANOVA results
	PRE	POST	PRE	POST	PRE	POST	*P* value, *F* value, η_*p*_^2^
DF ROM (°)	19.9 ± 7.3	27.8 ± 9.8*	20.5 ± 8.4	23.6 ± 9.8*	22.9 ± 9.9	22.4 ± 8.0	**T:** *p* < 0.01, *F* = 15.6, η_*p*_^2^ = 0.297
	*d* = 0.93		*d* = 0.36		*d* = −0.06		**G × T**: *p* < 0.01, *F* = 10.2, η_*p*_^2^ = 0.356
passive torque at DF ROM (Nm)	20.3 ± 7.3	26.2 ± 10.5*	22.8 ± 11.8	24.3 ± 13.0	22.6 ± 8.6	21.3 ± 7.7	**T:** *p* = 0.07, *F* = 3.5, η_*p*_^2^ = 0.086
	*d* = 0.64		*d* = 0.16		*d* = −0.14		**G × T**: *p* < 0.01, *F* = 5.4, η_*p*_^2^ = 0.227
first stretch sensation (°)	14.0 ± 15.1	15.4 ± 6.7	12.4 ± 6.7	11.4 ± 8.0	15.4 ± 9.1	15.8 ± 9.0	**T:** *p* = 0.28, *F* = 1.2, η_*p*_^2^ = 0.031
	*d* = 0.14		*d* = −0.10		*d* = 0.04		**G × T**: *p* = 0.57, *F* = 0.6, η_*p*_^2^ = 0.03
muscle stiffness (N/mm)	13.0 ± 9.0	5.6 ± 3.3*	12.1 ± 11.4	9.2 ± 8.0*	14.7 ± 8.8	14.3 ± 9.5	**T:** *p* < 0.01, *F* = 22.3, η_*p*_^2^ = 0.376
	*d* = −0.76		*d* = −0.30		*d* = −0.04		**G × T**: *p* < 0.01, *F* = 7.9, η_*p*_^2^ = 0.300

*d, effect size.*

*The two-way ANOVA results (T, time effect; G × T, group × time interaction effect; F-value) and partial η^2^ (η_p_^2^) are shown in right column.*

*, significant (p < 0.05) difference from the PRE value.

The Spearman’s rank correlation coefficients showed significant positive correlations between DF ROM and passive torque at DF ROM (*r*_*s*_ = 0.518, *p* < 0.01) or muscle stiffness (*r*_*s*_ = −0.603, *p* < 0.01) in HI-SS groups. However, there were no significant correlations between DF ROM and passive torque at DF ROM (*r*_*s*_ = 0.11, *p* = 0.72) or muscle stiffness (*r*_*s*_ = −0.126, *p* = 0.681) in LI-SS groups.

### Maximal Voluntary Isometric Contraction, Maximal Voluntary Concentric Contraction Torque, and Drop Jump Height

Changes in MVC-ISO, maximal voluntary concentric contraction (MVC-CON) torque, and drop jump height after the 4-week regular SS intervention program are shown in Table 3. There were no significant interaction effects or main effects of time in the any variable.

**TABLE 3:** Changes (mean ± SD) in maximal voluntary isometric contraction torque of plantar flexors (MVC-ISO) at three different positions, maximal voluntary concentric contraction torque (MVC-CON) at 30 and 120°/s and drop jump height before (PRE) and after (POST) 4-week static stretching program at high-intensity (HI) or low-intensity (LI), and control group without stretching intervention.

	HI stretching group		LI stretching group		Control group		ANOVA results
	PRE	POST	PRE	POST	PRE	POST	*P* value, *F* value, η_*p*_^2^
MVC-ISO at 30° plantar flexion (Nm)	52.5 ± 20.1	55.9 ± 17.6	54.8 ± 19.5	50.4 ± 20.0	61.4 ± 15.9	64.1 ± 16.3	**T:** *p* = 0.64, *F* = 0.23, η_*p*_^2^ < 0.01
	*d* = 0.18		*d* = −0.24		*d* = 0.15		**G × T**: *p* = 0.26, *F* = 1.41, η_*p*_^2^ = 0.07
MVC-ISO at neutral position (Nm)	146.9 ± 30.2	148.1 ± 22.0	146.6 ± 27.1	148.8 ± 28.9	170.8 ± 24.0	171.1 ± 19.4	**T:** *p* = 0.46, *F* = 0.55, η_*p*_^2^ = 0.02
	*d* = 0.04		*d* = 0.08		*d* = 0.01		**G × T**: *p* = 0.25, *F* = 0.25, η_*p*_^2^ = 0.01
MVC-ISO at 15° dorsiflexion (Nm)	193.2 ± 43.6	198.4 ± 29.2	191.0 ± 37.6	189.1 ± 42.9	191.4 ± 40.7	193.5 ± 45	**T:** *p* = 0.46, *F* = 0.55, η_*p*_^2^ = 0.02
	*d* = 0.13		*d* = −0.05		*d* = 0.05		**G × T**: *p* = 0.81, *F* = 0.21, η_*p*_^2^ = 0.01
MVC-CON at 30°/s (Nm)	115.2 ± 32.2	120.4 ± 21.9	116.8 ± 22.8	120.0 ± 26.4	131.7 ± 17.3	135.5 ± 15.2	**T:** *p* = 0.24, *F* = 1.46, η_*p*_^2^ = 0.04
	*d* = 0.22		*d* = 0.13		*d* = 0.16		**G × T**: *p* = 0.63, *F* = 0.48, η_*p*_^2^ = 0.03
MVC-CON at 120°/s (Nm)	73.6 ± 16.2	74.5 ± 15.0	74.2 ± 17.6	72.7 ± 20.0	68.2 ± 18.3	69.7 ± 18.5	**T:** *p* = 0.17, *F* = 1.98, η_*p*_^2^ = 0.05
	*d* = 0.05		*d* = −0.09		*d* = 0.09		**G × T**: *p* = 0.71, *F* = 0.35, η_*p*_^2^ = 0.02
drop jump height (cm)	18.6 ± 2.3	19.9 ± 3.2	17.0 ± 3.3	17.0 ± 3.7	20.2 ± 3.5	20.1 ± 3.3	**T:** *p* = 0.74, *F* = 0.11, η_*p*_^2^ < 0.01
	*d* = 0.43		*d* = 0.00		*d* = −0.03		**G × T**: *p* = 0.13, *F* = 2.21, η_*p*_^2^ = 0.11

*d, effect size.*

*The two-way ANOVA results (T: time effect, G × T: group × time interaction effect; F-value) and partial η^2^ (η_p_^2^) are shown in right column.*

### Muscle Architecture (Muscle Thickness, Pennation Angle, and Fascicle Length)

Changes in muscle architecture after 4 weeks of the regular SS intervention program are shown in Table 4. There were no significant interaction effects or main effects of time in any variable except for a main effect of time in muscle thickness in the LG. The *post hoc* test showed no significant changes in muscle thickness of the LG in any of the groups.

**TABLE 4:** Changes (mean ± SD) in muscle architecture (muscle thickness, pennation angle, and fascicle length) in medial gastrocnemius muscle (MG), lateral gastrocnemius muscle (LG), and soleus before (PRE) and after (POST) 4-week static stretching program at high-intensity (HI) or low-intensity (LI), and control group without stretching intervention.

	HI stretching group		LI stretching group		Control group		ANOVA results
	PRE	POST	PRE	PRE	POST	PRE	*P* value, *F* value, η_*p*_^2^
muscle thickness at MG (mm)	19.2 ± 2.9	19.5 ± 2.5	20.7 ± 2.5	20.5 ± 2.8	19.7 ± 3.0	19.4 ± 2.7	**T:** *p* = 0.20, *F* = 1.68, η_*p*_^2^ = 0.04
	*d* = 0.11		*d* = −0.07		*d* = −0.11		**G × T**: *p* = 0.11, *F* = 2.37, η_*p*_^2^ = 0.11
muscle thickness at LG (mm)	15.8 ± 2.4	15.5 ± 2.4	18.3 ± 1.8	17.5 ± 2.1	17.5 ± 2.6	17.5 ± 2.5	**T:** *p* = 0.02, *F* = 5.97, η_*p*_^2^ = 0.14
	*d* = −0.13		*d* = −0.35		*d* = 0.00		**G × T**: *p* = 0.13, *F* = 2.12, η_*p*_^2^ = 0.10
muscle thickness at soleus (mm)	17.7 ± 3.1	17.4 ± 3.3	19.4 ± 2.8	19.4 ± 3.1	19.6 ± 3.4	19.7 ± 2.6	**T:** *p* = 0.67, *F* = 0.19, η_*p*_^2^ < 0.01
	*d* = −0.10		*d* = 0.00		*d* = 0.03		**G × T**: *p* = 0.69, *F* = 0.38, η_*p*_^2^ = 0.02
pennation angle at MG (deg)	22.5 ± 1.9	22.0 ± 2.2	22.8 ± 3.1	21.3 ± 2.7	23.6 ± 2.9	22.6 ± 2.8	**T:** *p* = 0.07, *F* = 3.59, η_*p*_^2^ = 0.09
	*d* = −0.19		*d* = −0.57		*d* = −0.38		**G × T**: *p* = 0.70, *F* = 0.36, η_*p*_^2^ = 0.02
pennation angle at LG (deg)	13.4 ± 3.1	14.4 ± 2.4	15.1 ± 3.0	16.0 ± 2.6	15.2 ± 3.0	15.5 ± 1.8	**T:** *p* = 0.13, *F* = 2.43, η_*p*_^2^ = 0.06
	*d* = 0.33		*d* = 0.30		*d* = 0.10		**G × T**: *p* = 0.84, *F* = 0.171, η_*p*_^2^ < 0.01
fascicle length at MG (mm)	55.1 ± 9.5	54.5 ± 7.1	55.6 ± 6.1	53.8 ± 5.7	51.0 ± 7.1	53.8 ± 12.0	**T:** *p* = 0.88, *F* = 0.02, η_*p*_^2^ < 0.01
	*d* = −0.08		*d* = −0.24		*d* = 0.37		**G × T**: *p* = 0.20, *F* = 1.71, η_*p*_^2^ = 0.08
fascicle length at LG (mm)	62.7 ± 8.7	60.2 ± 7.6	59.0 ± 8.3	58.9 ± 7.1	60.1 ± 12.2	57.6 ± 14.8	**T:** *p* = 0.12, *F* = 2.61, η_*p*_^2^ = 0.07
	*d* = −0.26		*d* = −0.01		*d* = −0.26		**G × T**: *p* = 0.95, *F* = 0.05, η_*p*_^2^ < 0.01

*d, effect size.*

*The two-way ANOVA results (T: time effect, G × T: group × time interaction effect; F-value) and partial η^2^ (η_p_^2^) are shown in right column.*

## Discussion

In the present study, we investigated the effects of a SS intervention program with two different stretching intensities on the passive and active properties of the plantar flexor muscles. We showed that both HI-SS and LI-SS training programs increased the DF ROM and decreased muscle stiffness, however, results were significantly larger for HI-SS. In addition, only the HI-SS presented significant changes in stretch tolerance, as represented by the passive torque at DF ROM. Conversely, there were no significant changes in muscle strength or muscle architecture for both conditions. To the best of our knowledge, this is the first study to compare the effects of SS with different intensities on the passive and active properties of the plantar flexors, and it adds to previous works that showed the regular LI-SS training program increased DF ROM and decreased muscle stiffness of MG (Weppler and Magnusson, 2010; Nakamura et al., 2012, 2017, 2021a; Blazevich et al., 2014; Andrade et al., 2020).

In relation to DF ROM and muscle stiffness, the changes of larger magnitudes favoring the HI-SS occurred as we hypothesized. In fact, a previous acute study pointed out that stretching intensity could be an important factor in these adaptations (Apostolopoulos et al., 2015). As well as in a previous work of Nakamura et al. (2021a) we here observed that the changes in DF ROM were positively associated with the changes in passive torque at the DF ROM (*r* = 0.518; *p* < 0.001). Moreover, the changes in DF ROM were here significantly negatively associated with the changes in muscle stiffness (*r* = −0.603; *p* < 0.001). These results indicate that both changes in stretch tolerance and muscle stiffness contribute to an increase in the DF ROM (Nakamura et al., 2021b), and explain the associated larger benefits for HI-SS observed only on these three variables.

It is assumed that SS interventions at higher intensities could cause greater stretch stress on the muscle-tendon complex, leading to immediate changes in stretch tolerance and muscle stiffness (Fukaya et al., 2020a,b; Takeuchi and Nakamura, 2020a; Takeuchi et al., 2021a). In fact, it was recently demonstrated that high-intensity SS exercise can induce an immediate larger change in stretch tolerance (Freitas et al., 2015). With regard to SS training program, a previous study showed that the changes in stretch tolerance occur earlier adaptation than changes in muscle stiffness (Nakamura et al., 2017). As we did not investigate the time-course of the changes in these variables, future studies may consider exploring it to identify whether there are differences between HI-SS and LI-SS groups; i.e., whether different intensities influence stretch tolerance and muscle stiffness in different time-courses. Interestingly, our results are in opposition to those of Beltrão et al. (2020) and Fukaya et al. (2020b), which found no differences between low and high intensities on improving muscle passive properties. However, it is important to note that they analyzed the hamstring muscles, while we analyzed the plantar flexors. The effects of higher vs. lower intensities remain to be further investigated on different muscles. It is also necessary to investigate these concerns on subjects of different training backgrounds, sports practices, sex, and ages.

In parallel with our findings, a recent review indicated that most previous works also showed no significant changes in muscle architecture following SS training programs (Nunes et al., 2020b). The lack of effect seems to be related to the type of SS. Two main studies that showed some improvements on muscle strength and hypertrophy included an external overload to SS (Freitas and Mil-Homens, 2015; Simpson et al., 2017), whereas passive SS (without overload) may not induce significant changes, even when performing at a high protocol length (Moltubakk et al., 2021), or exercise volume (Longo et al., 2021), duration (Yahata et al., 2021), or intensity of discomfort (as the present work). Such findings contradict our hypothesis that HI-SS would be effective but, on the other hand, suggest that adding an external load to SS may be necessary to induce meaningful morphofunctional adaptations. More studies on this topic are needed.

From a practical point of view, an increase in ROM and/or decrease in muscle stiffness may be important for improving efficiency in some sports-related tasks and activities of daily living (Mulholland and Wyss, 2001; Hemmerich et al., 2006), as well as reducing the risk of muscle strain or injury (Witvrouw et al., 2003). With longer interventions, more apparent effects could have been noticed. Thus, HI-SS may be inserted in some sports settings as well as for the general population to reduce muscle stiffness. Some may be concerned about the increased rate of pain/discomfort applied during HI-SS; that is, subjects may not adhere to the intervention due to this. However, during the present study, no subject reported muscle soreness, and, in previous studies, it is indicated that if HI-SS results in some perceived muscular pain/discomfort, it may disappear within the next few hours (Takeuchi and Nakamura, 2020a,b). In addition, an active or passive warm-up could ameliorate the stretch tolerance (Takeuchi et al., 2021d), enabling the HI-SS to be performed.

There were some limitations in this study. First, we adopted the randomized control design in this study, and it is possible that the participants’ feelings and stretch tolerance could affect the results in the current study. Thus, further studies are needed to investigate the effects of participants’ feelings and stretch tolerance during the SS training program on ROM and muscle stiffness change with, e.g., a crossover design. We measured the muscle architecture at the rest position (Panidi et al., 2021), but there was a possibility that the ankle angle at the rest position could be changed after SS training program. Thus, measuring the muscle architecture at various ankle joint angles before and after the SS training program should be considered in future studies.

## Conclusion

In conclusion, performing HI-SS is more effective than LI-SS for increasing DF ROM and decreasing muscle stiffness following a 4-week training period in young men. However, SS performed in a stretching board with no additional external overload seems not to increase plantar flexors muscle strength or hypertrophy, regardless of the stretching discomfort intensity.

## Data Availability Statement

The raw data supporting the conclusions of this article will be made available by the authors, without undue reservation.

## Ethics Statement

The studies involving human participants were reviewed and approved by the Ethics Committee of the Niigata University of Health and Welfare, Niigata, Japan (Procedure #17677), and has complied with the Declaration of Helsinki requirements. The patients/participants provided their written informed consent to participate in this study.

## Author Contributions

MN contributed to study design and data collection, drafted the manuscript, and critically revised the manuscript. RY, SS, KY, YM, and KK contributed to data collection and made critical revisions to the manuscript. TF, KT, JN, and AK contributed to study design, data analysis, and made critical revisions to the manuscript. All authors approved the final version of the manuscript and agreed to be accountable for all aspects of the work.

## Conflict of Interest

The authors declare that the research was conducted in the absence of any commercial or financial relationships that could be construed as a potential conflict of interest.

## Publisher’s Note

All claims expressed in this article are solely those of the authors and do not necessarily represent those of their affiliated organizations, or those of the publisher, the editors and the reviewers. Any product that may be evaluated in this article, or claim that may be made by its manufacturer, is not guaranteed or endorsed by the publisher.
